# Prevalence, Awareness, Treatment, and Control of Hypertension in United States Counties, 2001–2009

**DOI:** 10.1371/journal.pone.0060308

**Published:** 2013-04-05

**Authors:** Casey Olives, Rebecca Myerson, Ali H. Mokdad, Christopher J. L. Murray, Stephen S. Lim

**Affiliations:** 1 Institute for Health Metrics and Evaluation, University of Washington, Seattle, Washington, United States of America; 2 Department of Biostatistics, University of Washington, Seattle, Washington, United States of America; 3 Harris School of Public Policy, University of Chicago, Chicago, Illinois, United States of America; University of Swansea, United Kingdom

## Abstract

Hypertension is an important and modifiable risk factor for cardiovascular disease and mortality. Over the last decade, national-levels of controlled hypertension have increased, but little information on hypertension prevalence and trends in hypertension treatment and control exists at the county-level. We estimate trends in prevalence, awareness, treatment, and control of hypertension in US counties using data from the National Health and Nutrition Examination Survey (NHANES) in five two-year waves from 1999–2008 including 26,349 adults aged 30 years and older and from the Behavioral Risk Factor Surveillance System (BRFSS) from 1997–2009 including 1,283,722 adults aged 30 years and older. Hypertension was defined as systolic blood pressure (BP) of at least 140 mm Hg, self-reported use of antihypertensive treatment, or both. Hypertension control was defined as systolic BP less than 140 mm Hg. The median prevalence of total hypertension in 2009 was estimated at 37.6% (range: 26.5 to 54.4%) in men and 40.1% (range: 28.5 to 57.9%) in women. Within-state differences in the county prevalence of uncontrolled hypertension were as high as 7.8 percentage points in 2009. Awareness, treatment, and control was highest in the southeastern US, and increased between 2001 and 2009 on average. The median county-level control in men was 57.7% (range: 43.4 to 65.9%) and in women was 57.1% (range: 43.0 to 65.46%) in 2009, with highest rates in white men and black women. While control of hypertension is on the rise, prevalence of total hypertension continues to increase in the US. Concurrent increases in treatment and control of hypertension are promising, but efforts to decrease the prevalence of hypertension are needed.

## Introduction

In the United States, high blood pressure is responsible for one in six deaths with one in three adults suffering from hypertension [1], [2], [3], [4]. Over the last two decades, national-level statistics show improvements in the awareness, treatment and control of hypertension; however, they remain far from ideal [5]. Among adults with hypertension, one in five remain unaware that they have hypertension, while half have not had their blood pressure controlled to levels less than 140/90 mmHg. There is likely to be even lower levels of awareness, treatment and control in different geographies in the United States. Data from the Behavioral Risk Factor Surveillance System (BRFSS) show large variation in the prevalence of self-reported hypertension across states. Other studies show substantially higher levels of hypertension in the South compared to other parts of the US [6].

Existing studies on geographic disparities in hypertension awareness and treatment suffer from at least one of two main limitations. The first is a reliance on self-report measures that miss individuals who are unaware that they have hypertension. Self-reported measurements also don’t allow one to discern those who are controlling their blood pressure through medication or other lifestyle modifications. The second limitation is an inability to report on geographical units smaller than the state-level. Health policy is increasingly being driven at the local levels and further progress in reducing the burden of high blood pressure in the United States will require a better understanding of, for example, the county-level distribution of interventions and their effectiveness. This type of disaggregated information generates awareness and can be used by states to compare the health of counties as a tool for targeting the use of scarce resources [7]. Additionally, the provision of local-level information allows for counties to advocate for investment in local programs using state, or potentially national funding sources [8], [9]. Lastly, tracking county-level trends can help to assess progress toward short-term or long-term health objectives [10].

In this study we address these two limitations by combining approaches for correcting self-report bias with small-area level estimation methods. We used National Health Examination and Nutrition Survey (NHANES) data to characterize the relationship between self-reported and physical measurements. We used the resulting model to predict physical measurements for BRFSS participants and employed small area estimation techniques to estimate hypertension prevalence, awareness, treatment, and control for adults aged 30 years and older by sex and race at the county-level.

## Methods

### Data Sources

The NHANES is a nationally representative cross-sectional survey of self-reported health and an extensive array of biomarkers. We used data from 1999–2008, which produces national-level estimates every two years. Additional information on the NHANES survey design is well documented elsewhere [11], [12]. The BRFSS is a state-level representative annual telephone survey that collects a range of self-reported measures [13]. This analysis used data from 1997–2009. Table S1 outlines the availability of key pieces of information on self-reported diagnosis and medication use for each of the years available in our dataset. We restricted our analysis to adults ages 30 years and older.

We extracted county-level race composition from National Center for Health Statistics (NCHS) population estimates, educational attainment from the 2000 Census, income and poverty estimates from the Census Bureau’s Small Area Income and Poverty Estimates, and number of fast food restaurants per 100k population from the Census Bureau’s County Business Patterns. In addition, we used county-level data from the 2009 Area Resource File on number of medical doctors and dentists per 100k population (Table S2) [14].

Our unit of analysis is the county. 3141 counties existed in 2009; however, the boundaries of counties changed between 2001 and 2009 [15]. We performed our analysis on 3133 counties, the largest set of constant counties from 2001 to 2009.

### Definitions

Total hypertension prevalence was defined as systolic BP of at least 140 mm HG and/or self-reported taking medication. We used standard definitions of hypertension awareness, treatment, and control (Table 1) [15], [16].

**Table 1:** Definitions of hypertension awareness, treatment, and control using self-reported diagnosis, self reported medication, and uncontrolled hypertension.

	Outcome			
Criteria	Awareness	Treatment	Control	
Have you ever been told by a doctor or other healthcare professionalthat you had hypertension?	Yes	Yes	Yes	
Because or your hypertension/high blood pressure, are you currentlytaking prescribed medication?	Yes or No	Yes	Yes	
Uncontrolled hypertension(SBP≥140 mm Hg)	Yes	Yes or No	No	
Population	SBP≥140 mm Hg and/or Treatment			

In both NHANES and BRFSS, race/ethnicity was determined by self-report and grouped as non-Hispanic white (white), non-Hispanic black (black), Hispanic, and other (American Indian, Native Alaskan, Asian or Pacific Islander, and other race not specified).

### Data Analysis

We used a two-stage approach to estimate the distribution of treatment and control of hypertension in US counties. Detailed information is provided in the online supplementary material; we briefly summarize the methods here. The first stage is designed to correct for self-report and diagnosis bias. Our approach is similar to those used in previous work to correct self-reported health data from the BRFSS, NHANES, and National Health Interview Survey [17], [18], [19], [20].

We constructed logistic regression models relating the probability of having uncontrolled hypertension to a set of covariates. Independent variables were chosen a priori to capture the effects of biological determinants, e.g. body mass index, socio demographic determinants, and the likelihood of individual contact with the health system and intervention use, e.g. current medication use. We stratified NHANES data by gender and by previous diagnosis, i.e. a total of 4 predictive logistic regression models [18]. The effect of medication among previously diagnosed individuals was allowed to be time-varying to reflect potential changes in treatment practices and efficacy. A summary of the individual predictor variables included in the first stage analysis and a comparison of their distributions in the NHANES and BRFSS are included in Table S3.

We used cross-validation to assess the predictive validity of the first-stage models by randomly holding out 20% of the NHANES data. For each of ten holdout samples, we measured the accuracy of predicting uncontrolled hypertensive status by calculating prediction accuracy (the proportion of correctly classified individuals).

The first-stage models were used to impute uncontrolled hypertensive status for individuals in the BRFSS by drawing from their respective posterior predictive distributions; we created ten imputed BRFSS datasets. For each of the datasets, individually imputed uncontrolled hypertensive status was combined with previous diagnosis and treatment variables to determine prevalence, awareness, treatment, and control of hypertension. We applied a grouped logistic spatio-temporal hierarchical regression model, stratifying by sex, which included dummy variables for age-group and race, as well as a set of county-level covariates, including demographic composition, education, income, poverty, and the number of limited-service restaurants, dentists, and medical doctors per 100,000 population (see Appendix S1 for more detail).

Model selection and validation was performed using the approach outlined by Srebotnjak et al wherein county-level predictions are validated against a pooled gold standard [21]. We used this approach to perform variable selection as well as to determine the likely performance of the small area models in sparsely populated counties. We calculated the root mean squared error (RMSE) and concordance correlation as a means of quantifying error. The final model was chosen on this basis.

Age-race -county-year predictions were weighted by the county-level race distribution using the 2003 NCHS population estimates to produce race-county-year predictions, and age standardized to the 2000 national age-distribution to provide county-year predictions. Final estimates reflect uncertainty from both stages of analysis [22], [23]. All data analysis was conducted using R software version 2.12.1 [24]. All reported survey estimates were calculated using the appropriate sampling weights and survey design.

## Results

### First-stage Regression and Validation

All regression results from the first stage model are shown in Table S4 and Table S5. For all regressions, age was positively and significantly associated with prevalence of uncontrolled hypertension, with the effect being non-linear as captured by the inclusion of a quadratic age term. Black men and women had significantly higher risk of uncontrolled hypertension compared to whites in both previously diagnosed and never diagnosed groups. Hispanics tended to have lower levels of uncontrolled hypertension than whites, except in the case of previously diagnosed men, where the effect was positive and statistically significant. BMI was significantly and positively associated with uncontrolled hypertension among never diagnosed men and women. Health insurance coverage was negatively associated with uncontrolled hypertension and reached statistical significance in 3 of 4 regression models. Among previously diagnosed men, self-reported medication use was negatively associated with uncontrolled hypertension.

First-stage cross-validation results suggest high prediction accuracy. The overall prediction accuracy for men and women was 0.73 (0.71–0.74) and 0.74 (0.71–0.75) for men and women, respectively. The prediction accuracy was lower for those with a previous diagnosis: 0.58 (0.56–0.62) for men and 0.56 (0.52–0.58) for women. Prediction accuracy was higher for never-diagnosed men and women: 0.81 (0.79–0.82) and 0.83 (0.80–0.85), respectively.

### Second Stage Model Validation

Small area models performed better for women than men, reflecting the difference in sex-specific sample size in the BRFSS (Figure S1). The concordance correlation between in-sample fits and the pooled gold standard were 81.6% and 92.7% in men and women, respectively. Likewise, the in-sample RMSE were 1.6% and 1.4% for men and women, respectively. In comparison, single-year direct estimates had concordance correlation of less than 25% with the pooled gold standard and exhibited RMSE greater than 9%.

Our model was robust to small sample sizes. In counties with as few as 10 observations in a given year, we estimated correlation and RMSE of 65.4% and 2.3%, respectively, in men and 83.5% and 2.4%, respectively, in women. In contrast, single-year direct estimates had a correlation of less than 3% and RMSE of greater than 20%.

### Self-reported, Total, and Uncontrolled Hypertension

In 2009, the median county prevalence of self-reported hypertension was 37.0% (range: 18.2 to 61.2%) in men and 34.7% (range: 16.8 to 48.8%) in women (Table 2). After correcting for self-reporting bias, median county prevalence of total hypertension was 37.6% and 40.1% in men and women, respectively, in 2009 (0.56 and 5.33 percentage points higher than self-reported prevalence). This is consistent with previous findings suggesting that self-reported hypertension is more correlated with clinical hypertension in women than in men [18]. The median country prevalence of self-reported and total hypertension increased over the study period in both men and women.

**Table 2:** Age-standardized median and range of county-level self-reported prevalence, total prevalence, awareness, treatment, and control of hypertension by sex and race in adults 30 years and older in 2001 and 2009.

	Self-Report		Prevalence		Awareness		Treatment		Control	
	2001	2009	2001	2009	2001	2009	2001	2009	2001	2009
**Men**	31.46	36.98	32.58	37.56	78.16	82.36	64.96	73.05	47.25	57.69
	(18.83−46.59)	(18.22−61.16)	(23.56−47.25)	(26.53−54.43)	(65.04−84.13)	(70.31−88.08)	(44.74−75.03)	(55.04−82.01)	(32.03−55.49)	(43.42−65.86)
White	31.18	35.84,	32.35	37.23	78.08	81.93	64.17	72.29	49.32	58.63
	(19.05−48.16)	(22.19−54.15)	(23.75−40.93)	(26.83−46.95)	(63.66−84.79)	(67.56−87.82)	(41.72−74.75)	(51.04−82.23)	(29.89−58.1)	(39.37−66.31)
Black	43.33	48.46	45.57	50.84	78.61	82.03	64.73	72.24	47.07	55.68
	(28.74−61.02)	(32.73−66.61)	(34.94−54.97)	(38.67−60.92)	(65.15−84.82)	(68.68−87.55)	(43.41−74.78)	(52.19−81.77)	(29.24−54.96)	(38.12−62.7)
Hispanic	30.94	35.59	33.65	38.13	76.3	79.87	58.99	67.41	40.95	50.46
	(18.87−47.89)	(21.99−53.88)	(25.03−42.10)	(27.72−47.70)	(62.92−83.27)	(65.89−86.23)	(37.21−70.32)	(45.86−78.67)	(23.37−49.83)	(31.84−58.84)
Other	34.42	39.26	36.48	41.39	76.24	80.12	61.7	69.86	45.87	55.1
	(21.5−51.79)	(24.89−57.74)	(27.27−45.29)	(30.43−51.22)	(61.92−83.25)	(65.57−86.36)	(39.63−72.52)	(48.56−80.28)	(27.26−54.61)	(36.22−62.95)
**Women**	31.48	34.75	36.94	40.08	76.73	80.25	67.56	74.08	43.83	57.06
	(17.78−51.73)	(16.77−58.67)	(26.75−52.97)	(28.52−57.88)	(61.86−87.69)	(65.71−90.26)	(50.87−81.53)	(57.68−86.43)	(30.86−53.48)	(43.04−65.46)
White	30.71	33.25	35.69	38.85	78.05	81.54	68.95	75.53	43.2	57.93
	(15.33−46.67)	(16.67−48.79)	(26.6−42.95)	(28.35−48.01)	(63.24−85.06)	(67.09−88.32)	(51.62−78.76)	(59.06−84.53)	(29.78−53.46)	(43.98−67.66)
Black	50.19	53.05	50.6	54.39	88	89.86	80.32	84.8	50.46	63.7
	(30.26−66.54)	(32.26−68.47)	(39.16−58.89)	(41.85−64.18)	(78.21−91.85)	(80.92−93.37)	(67.44−86.65)	(73.72−90.13)	(39.55−58.13)	(54.29−69.86)
Hispanic	35.13	37.81	39.19	42.64	78.36	81.88	69.11	75.8	45.23	59.53
	(18.35−51.56)	(19.87−53.68)	(29.58−46.7)	(31.68−51.98)	(63.82−85.21)	(67.89−88.39)	(51.96−78.82)	(59.76−84.53)	(31.61−55.32)	(45.81−68.76)
Other	36.08	38.78	42.66	46.03	73.76	77.76	65.12	71.98	42.16	56.10
	(19.03−52.57)	(20.58−54.69)	(33.19−50.01)	(35.27−55.16)	(58.22−81.62)	(62.46−85.38)	(47.55−75.51)	(55.03−81.64)	(28.48−52.52)	(41.77−65.98)

**Figure 1:**
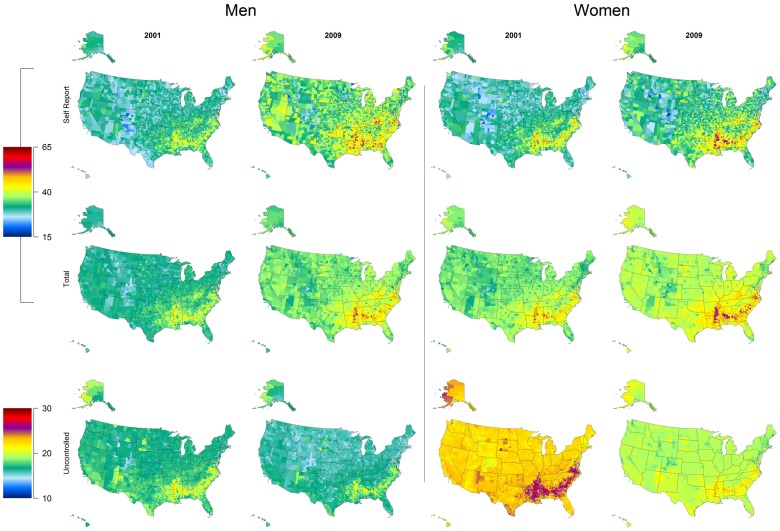
Age-standardized prevalence of self-reported, total, and uncontrolled hypertension by sex among adults 30 years and older in 2001 and 2009.

Total hypertension prevalence exhibited strong geographic trends, with the heaviest burden localized to southeastern states (Figure 1). A handful of Colorado counties consistently had the lowest prevalence of total hypertension. These trends were even more pronounced when considering prevalence of uncontrolled hypertension (Figure 1), where counties with elevated burden were almost entirely localized to southeastern states, with the exception of a few counties in the Four Corners area and in South Dakota. The prevalence of uncontrolled hypertension decreased over the study period.

The spread of county-level uncontrolled hypertension prevalence within-states varied widely, ranging from 0.3 (Hawaii) to 7.8 (Virginia) percentage points in men and from 0.4 (Delaware) to 5.8 (Virginia) percentage points in women in 2009 (Figure S2). The states that experienced the largest geographic disparity by sex were Alabama, Mississippi, Georgia and Virginia. Among men, New Mexico, South Dakota, and North Dakota experienced geographic disparities on par with southern states.

**Figure 2:**
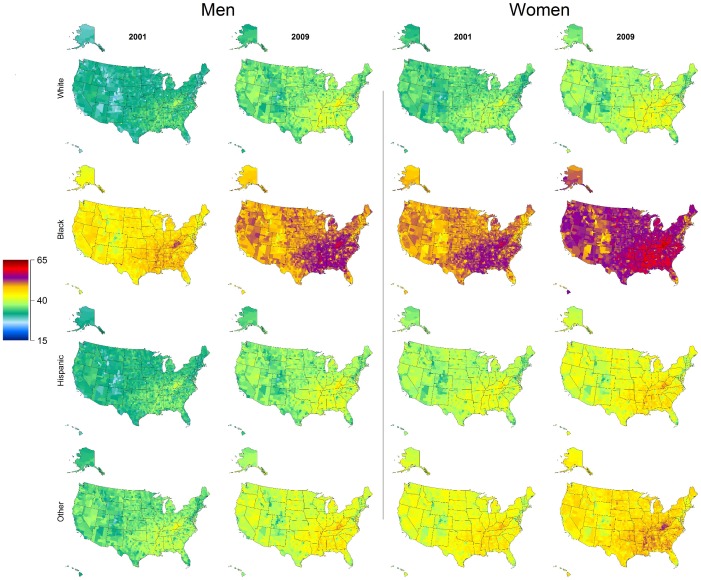
Age-standardized prevalence of total hypertension by sex and race among adults 30 years and older in 2001 and 2009.

Black men and women had the highest total prevalence of hypertension, with median prevalence of 50.8% (range: 38.7% to 60.9%) in men and 54.4% (range 41.8 to 64.2%) in women aged 30 and older in 2009. Median county prevalence of hypertension increased in both genders and all race categories between 2001 and 2009. Geographic trends in total prevalence were similar across race categories (Figure 2).

### Awareness, Treatment, and Control of Hypertension

Estimates of county-level awareness, treatment, and control by sex and race are provided in Table 2. Median awareness of hypertension in 2009 was 82.4% (range: 70.31 to 88.1%) in men and 80.3% (range: 65.7 to 90.3%) in women. Black men and women in 2009 tended to have the highest awareness, with county medians of 82.0% (range: 68.7 to 87.6%) and 89.9% (range 80.9 to 93.4), respectively. The counties with the lowest awareness were largely located in New Mexico and Colorado (Figure S3).

Median county-treatment levels in 2009 were 73.1% (range: 55.0 to 82.0%) in men and 74.1% (range: 57.7 to 86.4%) in women. The median percentage of untreated individuals who were aware of their hypertensive status decreased by 3.9 and 3.0 percentage points in men and women, respectively, between 2001 and 2009. There remained wide geographic disparities in county treatment levels within states in 2009 (Figure S2). For example, only 3% more men were treated in the county with the highest compared to the lowest treatment rates in Rhode Island, whereas in North Dakota the best-off and worst-off counties differed in their treatment of hypertension by more than 38%. For women, treatment rates differed in the best-off and the worst-off counties in Colorado and Nebraska by nearly 20 percentage points.

Treatment tended to be highest in black men and women, with median values of 72.2% (range: 52.2 to 81.8%) and 84.8% (range: 73.7 to 90.1%), respectively. However, the geographic distribution of treatment was the same for all races with the highest treatment levels in many southeastern states, and with lowest levels in Texas, New Mexico, and Colorado (Figure S4).

**Figure 3:**
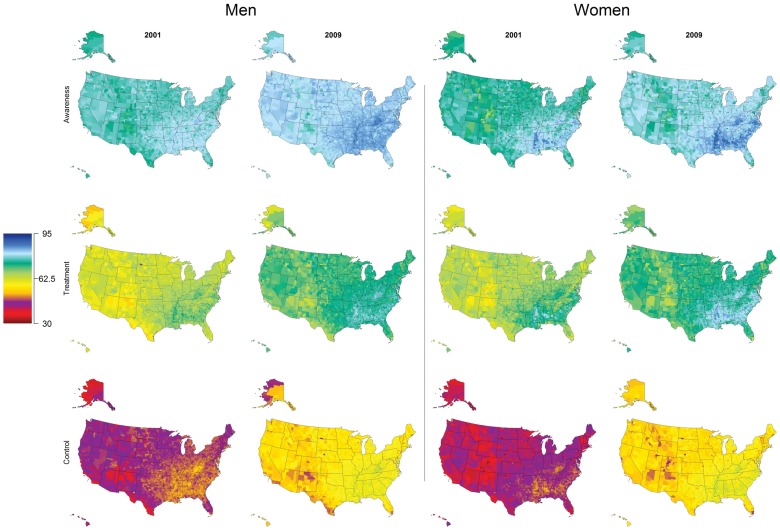
Age-standardized awareness, treatment, and control of hypertension by sex in adults 30 years and older in 2001 and 2009.

The median county-level control in men was 57.7% (range: 43.4 to 65.7%) and in women was 57.1% (range: 43.0 to 65.5%) in 2009. On average, 79% of treated men and 77% of treated women were controlled in 2009. The highest levels of control were generally localized to the southeastern states (Figure 3). In men, the lowest control was found in counties along the US-Mexico border in Texas and in New Mexico and Arizona, where as in women, low control was also evident in a number of counties in Colorado.

White men had the highest median county control levels, with a 57.7% (range: 43.4 to 65.9%) in men. However, black women had significantly higher control that white women, with a county median of 63.7% (range: 54.3 to 69.9%) in 2009. Across race categories, control was highest in the southeastern states (Figure S5).

## Discussion

To our knowledge, this work produces the first county-level estimates of hypertension prevalence, awareness, treatment, and control. Our approach builds on previous work in this area by employing sophisticated statistical methods for small area estimation and multiple imputation while maintaining a high degree of rigor through use of cross-validation for every step in the analysis.

Increasing trends in hypertension awareness, treatment, and control suggest that campaigns to increase hypertension awareness and treatment have been successful. Furthermore, prevalence of uncontrolled hypertension is on the decline. Likewise, in many cases, the within-state range of control decreased over the study period. Yet, with few exceptions, the range of total hypertension prevalence within any given state increased between 2001 and 2009. Thus, even though there is strong evidence to suggest that the Healthy People 2010 goal regarding control of hypertension is on track to being met in most counties, many counties are falling short of the overarching goal of reducing total prevalence [10].

We found high treatment levels in southern counties, where there tends to be an elevated risk of cardiovascular disease and death due to stroke [25]. Upon examining the prevalence of self-reported medication use, we found that in certain high-prevalence counties in the south, almost 50% of individuals reported taking antihypertensive medication in 2009. Given the high levels of SBP and heavy burden of diabetes, obesity, and other cardiovascular risk factors in southern states [6], [18], [21], [26], as well as research suggesting that treatment of hypertension is higher in those with co-morbid cardiovascular conditions [27], this finding could suggest a tendency on the part of physicians to treat individuals with high risk profiles more readily than in other parts of the country.

Recent research suggests that the high levels of uncontrolled hypertension in the US could be due to the failure to add antihypertensive medications when blood pressure remains uncontrolled using first or second line treatments [28], [29]. While we estimate high treatment of medication in many high prevalence counties, this works emphasizes that treatment practices are not often adequate to ensure control.

In many cases, counties with the lowest prevalence of hypertension tended to have low treatment and less control. The perception of good health can negatively impact both treatment and control of hypertension [30]. Counties with low prevalence of hypertension may also have lower mean SBP, which could influence treatment rates [18]. Consistent with a body of literature, our analysis did not distinguish between healthy individuals and those controlling their hypertension through lifestyle modifications [5], [18], [31], [32], [33]. The fact that the healthiest counties tended to be the least medicated, and oftentimes the least controlled, could reflect a tendency for these individuals to combat hypertension through lifestyle interventions.

Differences between NHANES and BRFSS survey design and geographical coverage contributes to differences in estimates arising from each survey. We compared the national estimates of uncontrolled hypertension in men and women stratified by previous diagnosis in NHANES and corrected BRFSS data over the study period to determine the extent of discordance at the national-level. In most cases, there was little evidence of statistically significant differences as evidenced by overlapping confidence intervals (Table S6).

The NHANES does not make geographic identifiers of its participants available to the public. Thus, we were unable to include geography into the first-stage bias correction. In order to investigate possible extra variation due to geography, we fit a mixed effects regression model to the NHANES data with a random intercept for NHANES sampling stratum, which are geographically diverse to ensure national representativeness. Stratum random intercepts did not show significant variation in any diagnosis-gender group (Figure S6), indicating that geographic variation beyond the individual covariates included in the correction model was minimal. This result is consistent with findings by Ezzati et al, who found that an individual-level bias correction adequately reflected geographic variation in diagnosis bias [18].

The geographic distribution of hypertension treatment and control is an important piece of information to inform public health policy and surveillance. Likewise, local-level information regarding hypertension prevalence and awareness should spur medical professionals to adhere more stringently to national treatment guidelines. Lastly, county-level information can empower the public to act. Ultimately, applying these methods to other cardiovascular risk factors will allow us to understand and evaluate the performance of local health systems with the ultimate goal of learning from successful programs and improving the efficiency of others.

## Supporting Information

Figure S1 **Root mean squared error (RMSE) and concordance correlation (CORR) between Small Area Model predictions (1997–2005) and pooled direct gold standard (2001–2009) of self-reported hypertension.**
(TIF)

Figure S2 **Within-state disparities in uncontrolled hypertension, and awareness, treatment, and control of hypertension by sex in adults ages 30 years and older in 2009.**
(TIF)

Figure S3 **Age-standardized awareness of hypertension by sex and race among adults 30 years and older in 2001 and 2009.**
(TIF)

Figure S4 **Age-standardized treatment of hypertension by sex and race in adults 30 years and older in 2001 and 2009.**
(TIF)

Figure S5 **Age-standardized control of hypertension by sex and race in adults 30 years and older 2001 and 2009.**
(TIF)

Figure S6 **Stratum-specific random intercepts stratified by sex and previous diagnosis in first stage bias-correction models.**
(TIF)

Table S1 **Availability of Diagnosis and Treatment Information for BRFSS 1997–2009.**
(DOCX)

Table S2 **Summary of county-level covariates used in second-stage small area model.**
(DOCX)

Table S3 **Summary of variables included in first stage analysis and their distributions in US adults ages 30 years and older in the NHANES and the BRFSS.**
(DOCX)

Table S4 **Coefficients for NHANES Predictive Models, Never Diagnosed Men and Women.**
(DOCX)

Table S5 **Coefficients for NHANES Predictive Models, Previously Diagnosed Men and Women.**
(DOCX)

Table S6 **Uncontrolled hypertension prevalence in NHANES and (imputed) BRFSS surveys by year and sex, age-standardized to 2000 US Population with 95% confidence intervals.**
(DOCX)

Appendix S1 (DOCX)
