# Supplementary figures and images for: Prevalence, Awareness, Treatment, and Control of Hypertension in United States Counties, 2001–2009
Source: PLoS One. 2013 Apr 5;8(4):e60308. doi: 10.1371/journal.pone.0060308 (PMC3618269; doi:10.1371/journal.pone.0060308)

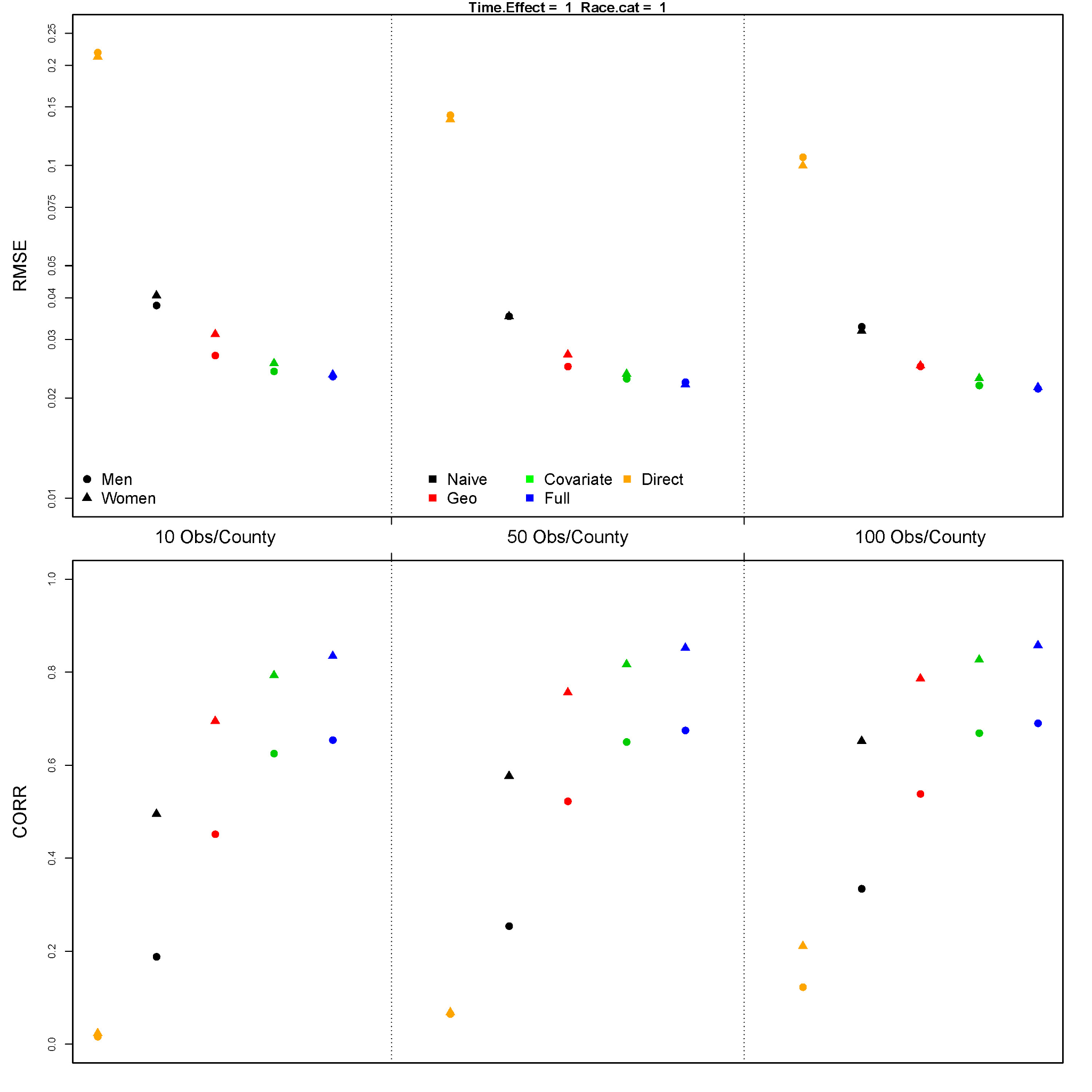

Supplement: Figure S1 — Root mean squared error (RMSE) and concordance correlation (CORR) between Small Area Model predictions (1997–2005) and pooled direct gold standard (2001–2009) of self-reported hypertension. (TIF) [file pone.0060308.s001.tif]

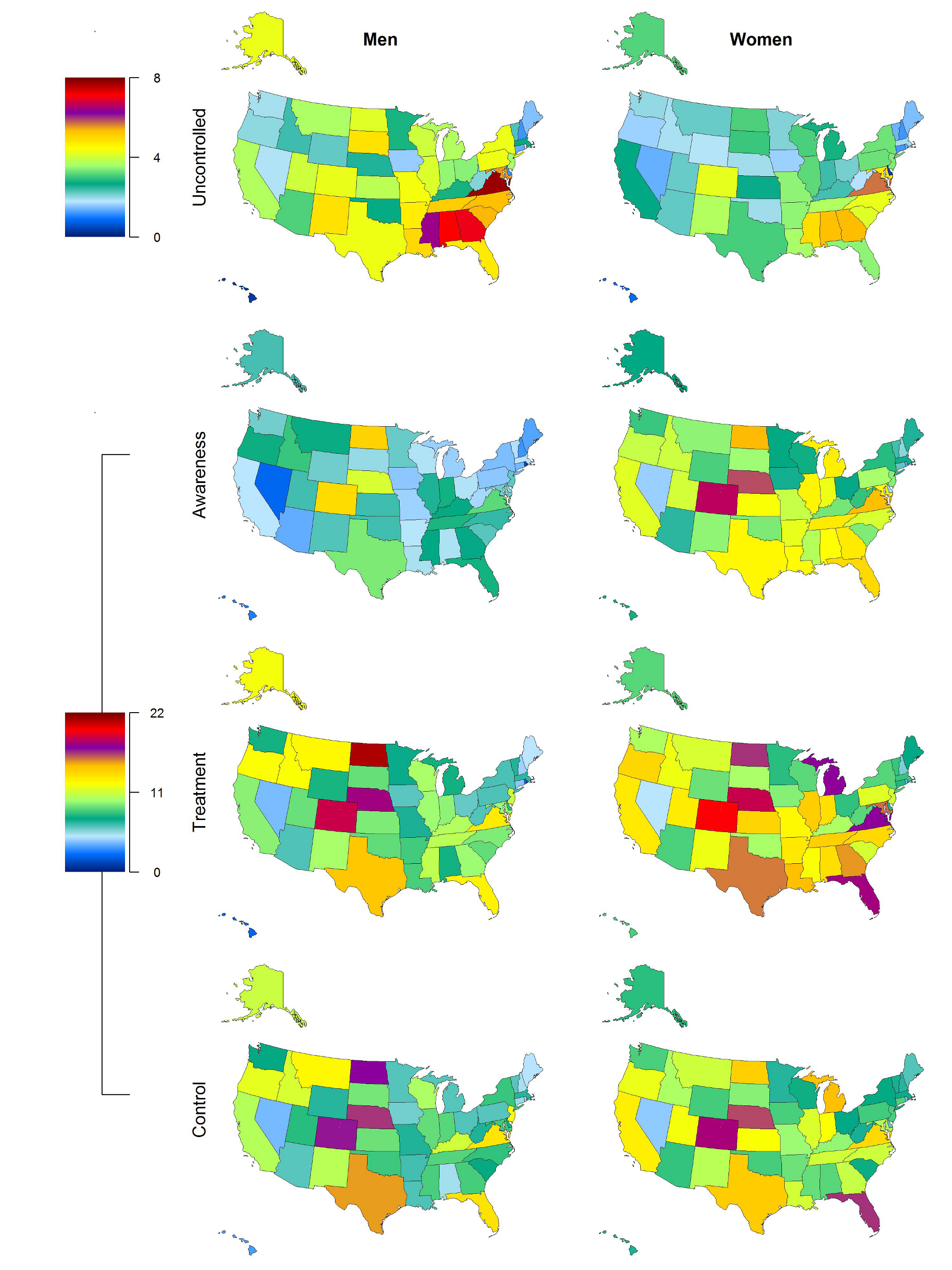

Supplement: Figure S2 — Within-state disparities in uncontrolled hypertension, and awareness, treatment, and control of hypertension by sex in adults ages 30 years and older in 2009. (TIF) [file pone.0060308.s002.tif]

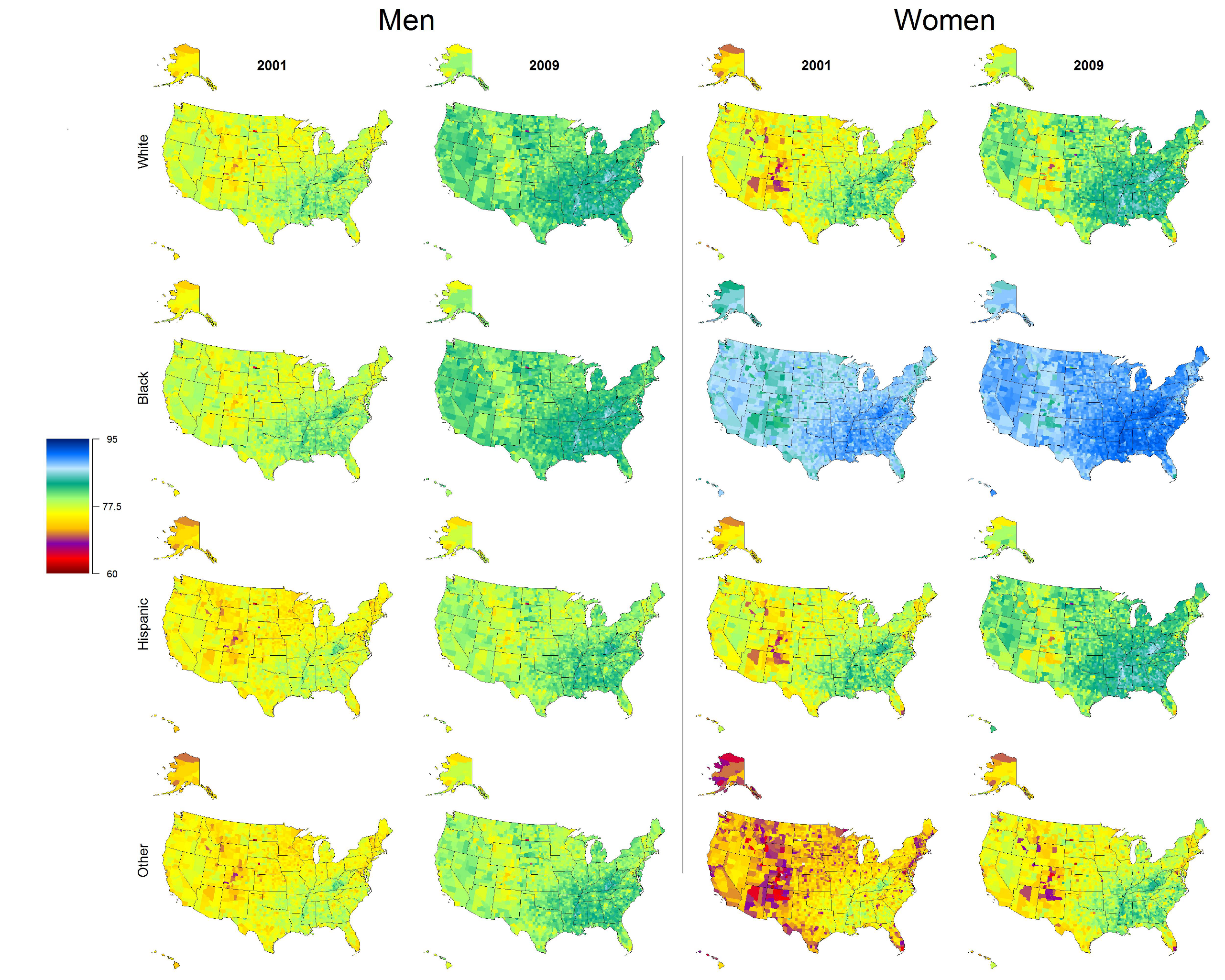

Supplement: Figure S3 — Age-standardized awareness of hypertension by sex and race among adults 30 years and older in 2001 and 2009. (TIF) [file pone.0060308.s003.tif]

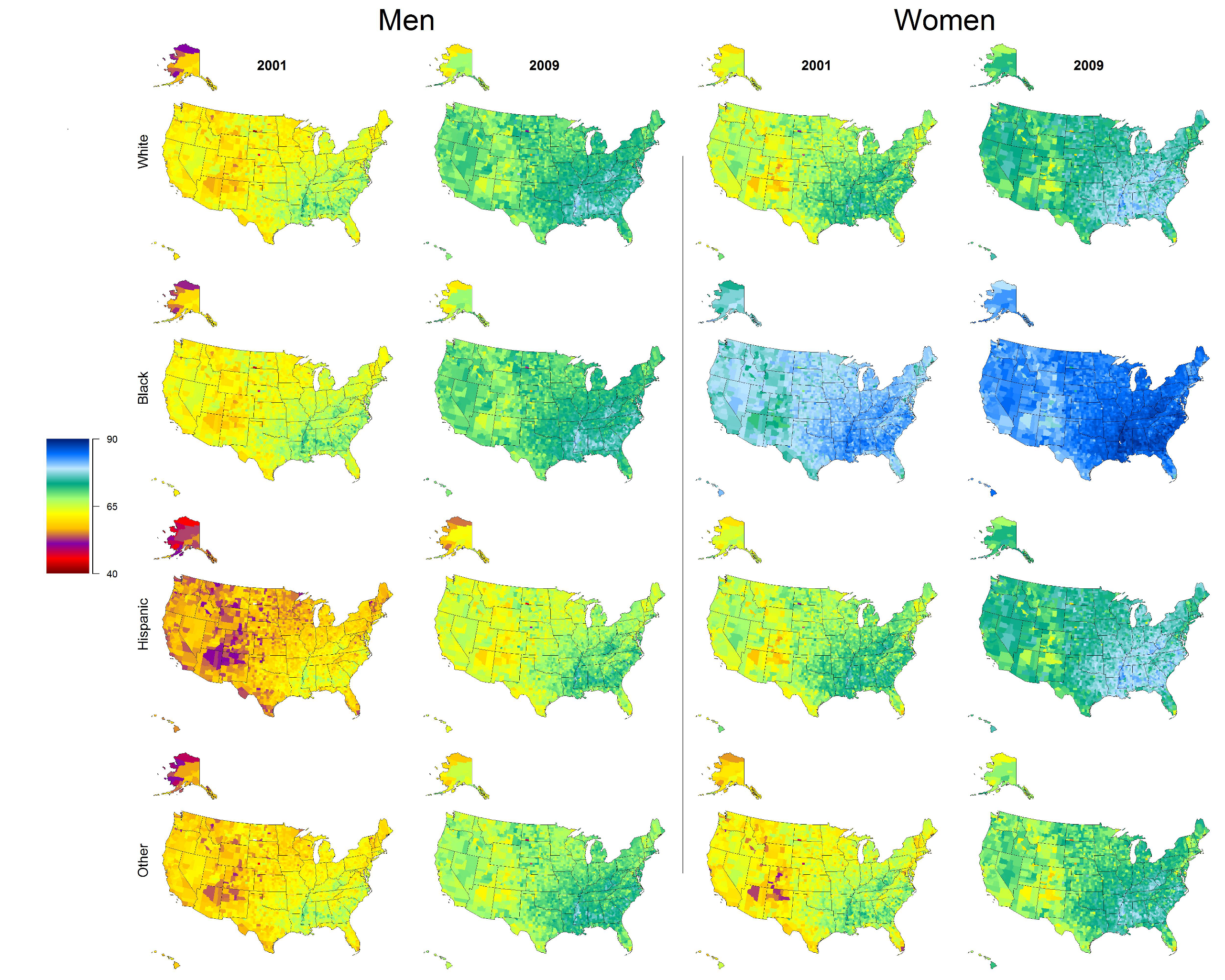

Supplement: Figure S4 — Age-standardized treatment of hypertension by sex and race in adults 30 years and older in 2001 and 2009. (TIF) [file pone.0060308.s004.tif]

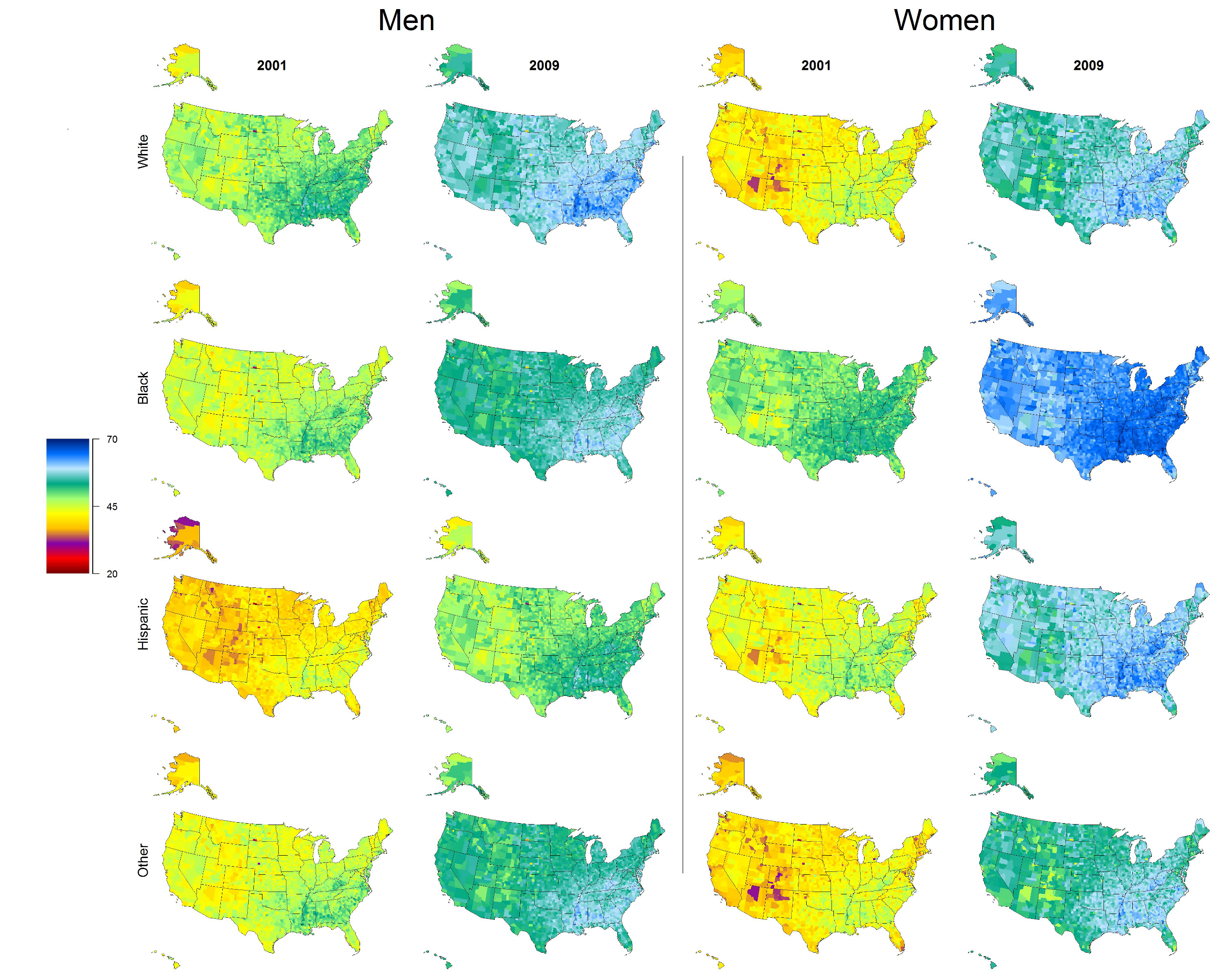

Supplement: Figure S5 — Age-standardized control of hypertension by sex and race in adults 30 years and older 2001 and 2009. (TIF) [file pone.0060308.s005.tif]

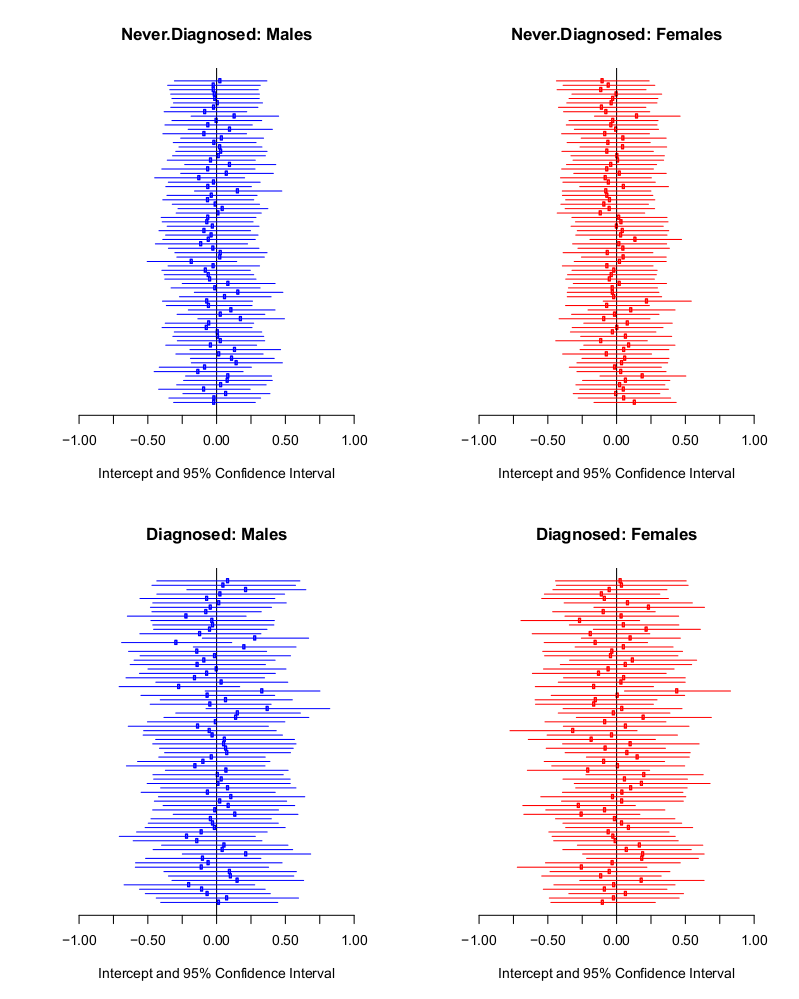

Supplement: Figure S6 — Stratum-specific random intercepts stratified by sex and previous diagnosis in first stage bias-correction models. (TIF) [file pone.0060308.s006.tif]
