# Supplementary material for: Prevalence, Awareness, Treatment, and Control of Hypertension in United States Counties, 2001–2009
Source: PLoS One. 2013 Apr 5;8(4):e60308. doi: 10.1371/journal.pone.0060308 (PMC3618269; doi:10.1371/journal.pone.0060308)
Supplement: Table S1 — Availability of Diagnosis and Treatment Information for BRFSS 1997–2009. (DOCX) [file pone.0060308.s007.docx]

Table S1: Availability of Diagnosis and Treatment Information for BRFSS 1997-2009.

|  | “Have you ever been told by a health professional that you have high blood pressure?” | | “Are you currently taking medication for your high blood pressure?” | |
| --- | --- | --- | --- | --- |
| Year | Men | Women | Men | Women |
| 1997 | 41989 | 58512 | NA* | NA |
| 1998 | 9879 | 13788 | NA | NA |
| 1999 | 48922 | 69341 | NA | NA |
| 2000 | 5696 | 8404 | NA | NA |
| 2001 | 64182 | 89460 | 64182 | 89460 |
| 2002 | 13231 | 18768 | 13231 | 18768 |
| 2003 | 83258 | 121637 | 83258 | 121637 |
| 2004 | 15657 | 25072 | 15657 | 25072 |
| 2005 | 112531 | 172057 | 112531 | 172057 |
| 2006 | NA | NA | NA | NA |
| 2007 | 130487 | 206611 | 130487 | 206611 |
| 2008 | NA | NA | NA | NA |
| 2009 | 130829 | 205062 | 130829 | 205062 |
| Total | 656661 | 991712 | 627061 | 948049 |

*Hypertension Awareness module not asked in any state during survey year.
