# Supplementary material for: Prevalence, Awareness, Treatment, and Control of Hypertension in United States Counties, 2001–2009
Source: PLoS One. 2013 Apr 5;8(4):e60308. doi: 10.1371/journal.pone.0060308 (PMC3618269; doi:10.1371/journal.pone.0060308)
Supplement: Table S2 — Summary of county-level covariates used in second-stage small area model. (DOCX) [file pone.0060308.s008.docx]

Table S2: Summary of county-level covariates used in second-stage small area model.

| Variable | Years Available | Source |
| --- | --- | --- |
| County Race/Ethnicity (%) | 1997-2009 | NCHS Bridged-Race population estimates (2007 vintage) |
| Non-Hispanic black |  |  |
| Non-Hispanic Asian |  |  |
| Non-Hispanic American Indian |  |  |
| Hispanic |  |  |
| Education (%) | 2000 | 2000 Census |
| High School degree |  |  |
| Bachelor’s degree |  |  |
| Poverty (%) | 1997-2009 | Census Bureau Small Area Income and Poverty Estimates |
| Household Income (CPI adjusted) | 1997-2009 | Census Bureau Small Area Income and Poverty Estimates |
| Number of Fast food restaurants per 100,000 population* | 1997-2009 | Census Bureau County Business Patterns |
| Number of medical doctors per 1,000 population | 2000 | Area Resource File |
| Number of dentists per 1,000 population | 2000 | Area Resource File |

*Time series was adjusted to address structural due to transition from SIC to NAICS and NAICS revisions and missing data were multiply imputed with auxiliary information and weak priors using R package Amelia (Srebotnjak et al). At the time of analysis, 2009 data were not available. Therefore, 2008 data are used for 2009 as well.
