# Supplementary material for: Prevalence, Awareness, Treatment, and Control of Hypertension in United States Counties, 2001–2009
Source: PLoS One. 2013 Apr 5;8(4):e60308. doi: 10.1371/journal.pone.0060308 (PMC3618269; doi:10.1371/journal.pone.0060308)
Supplement: Table S3 — Summary of variables included in first stage analysis and their distributions in US adults ages 30 years and older in the NHANES and the BRFSS. (DOCX) [file pone.0060308.s009.docx]

Table S3: Summary of variables included in first stage analysis and their distributions in US adults ages 30 years and older in the NHANES and the BRFSS*.

|  |  |  | NHANES 2-Year Block | | | | | BRFSS Year** | | | | | |
| --- | --- | --- | --- | --- | --- | --- | --- | --- | --- | --- | --- | --- | --- |
| Characteristics | Reason for Inclusion | Possible Value | 1999-2000 (n=5,239) | 2001-2002 (n=5,646) | 2003-2004 (n=5,584) | 2005-2006 (n=4,489) | 2007-2008 (n=5,391) | 1999 (n=130,542) | 2001 (n=183,156) | 2003 (n=242,019) | 2005 (n=279,557) | 2007 (n=332,814) | 2009 (n=327,159) |
| Uncontrolled Hypertension, % | Outcome variable | 1 (SBP ≥ 140 mm Hg), 0 (SBP < 140 mm Hg) | 20.4 (0.9) | 18.7 (0.9) | 17.9 (0.9) | 17.0 (1.0) | 15.8 (0.7) | NA ƚ | NA | NA | NA | NA | NA |
| Self Reported Hypertension, % | Stratification Variable | 1 (Yes). 0 (No) | 30.2 (1.0) | 31.1 (1.2) | 36.0 (1.2) | 35.1 (1.2) | 35.9 (1.2) | 29.5 (0.22) | 31.3 (0.20) | 31.3 (0.18) | 31.6 (0.16) | 32.7 (0.15) | 34.0 (0.14) |
| Sex, % | Stratification Variable | 1 (Female), 0 (Male) | 52.7 (0.81) | 51.9 (0.77) | 52.1 (0.70) | 52.2 (0.80) | 52.0 (0.65) | 52.0 (0.25) | 51.5 (0.22) | 51.1 (0.21) | 51.1 (0.19) | 51.1 (0.18) | 50.9 (0.17) |
| Self Reported Medication, % | Indicator of Hypertension control | 1 (Yes), 0 (No) | 23.2 (0.9) | 23.3 (1.0) | 27.3 (1.0) | 27.0 (1.0) | 28.4 (1.0) | NAǂ | 23.7 (0.17) | 24.8 (0.16) | 25.4 (0.14) | 27.0 (0.13) | 27.7 (0.12) |
| Mean age, yr | Predictor of Hypertension | 35-85 | 51.97 (7.2) | 51.92 (8.2) | 51.95 (6.8) | 52.13 (5.5) | 52.02 (5.4) | 51.9 (0.02) | 52.0 (0.01) | 52.0 (0.01) | 52.0 (0.01) | 52.0 (0.01) | 51.9 (0.01) |
| Race/  ethnicity, % | Predictor of Hypertension and health care access | White | 73.9 (3.8) | 75.8 (3.1) | 75.3 (3.6) | 74.6 (3.3) | 72.3 (3.9) | 78.5 (0.23) | 75.7 (0.23) | 75.0 (0.21) | 74.2 (0.20) | 73.1 (0.19) | 72.6 (0.18) |
|  |  | Black | 10.3 (1.9) | 10.1 (2.0) | 10.5 (2.1) | 10.9 (2.2) | 10.8 (2.2) | 9.0 (0.14) | 9.0 (0.13) | 9.2 (0.13) | 9.2 (0.15) | 8.9 (0.11) | 9.7 (0.12) |
|  |  | Hispanic | 11.9 (2.8) | 10.1 (2.2) | 9.3 (2.1) | 9.3 (1.8) | 11.2 (2.3) | 8.9 (0.18) | 9.4 (0.19) | 9.9 (0.18) | 10.7 (0.17) | 11.2 (0.17) | 11.4 (0.14) |
|  |  | Other | 3.9 (0.9) | 4.0 (0.7) | 4.9 (0.7) | 5.2 (0.7) | 5.8 (1.0) | 3.6 (0.11) | 5.8 (0.13) | 6.0 (0.12) | 6.0 (0.11) | 6.7 (0.12) | 6.3 (0.10) |
| Mean BMI, kg/m^2^ | Predictor of Hypertension and indicator for lifestyle factors | >0 | 27.7 (0.2) | 27.6 (0.2) | 27.9 (0.2) | 28.2 (0.2) | 28.1 (0.2) | 26.7 (0.02) | 27.0 (0.02) | 27.2 (0.02) | 27.4 (0.02) | 27.7 (0.02) | 27.8 (0.02) |
| Education, % | Indicator of HTN knowledge | Less Than HS | 25.5 (1.8) | 18.9 (1.6) | 18.7 (1.9) | 16.8 (1.1) | 20.3 (1.6) | 13.2 (0.18) | 12.6 (0.18) | 11.0 (0.15) | 10.7 (0.14) | 10.1 (0.14) | 9.4 (0.12) |
|  |  | HS Diploma | 26.6 (2.7) | 25.0 (1.3) | 27.1 (1.2) | 25.6 (1.4) | 25.3 (1.8) | 32.7 (0.23) | 30.8 (0.20) | 30.2 (0.16) | 29.1 (0.16) | 27.6 (0.16) | 26.7 (0.15) |
|  |  | More Than HS | 48.0 (2.8) | 56.1 (2.3) | 54.2 (1.8) | 57.6 (1.7) | 54.4 (2.6) | 54.1 (0.25) | 56.6 (0.22) | 58.9 (0.21) | 60.2 (0.19) | 62.2 (0.18) | 63.9 (0.17) |
| Health Insurance, % | Indicator of Hypertension knowledge and control | 1 (Yes), 0 (No) | 85.9 (1.6) | 87.7 (1.3) | 85.8 (1.4) | 86.0 (1.5) | 84.7 (1.2) | 89.5 (0.15) | 89.5 (0.15) | 88.3 (0.14) | 88.3 (0.13) | 88.6 (0.13) | 88.2 (0.13) |
| Smoker, % # | Predictor of Hypertension and indicator of lifestyle factors | 1 (Yes), 0 (No) | 51.9 (1.6) | 50.8 (1.7) | 52.2 (1.0) | 49.8 (1.8) | 47.8 (1.4) | 49.8 (0.2) | 50.5 (0.2) | 50.0 (0.2) | 47.8 (0.2) | 46.0 (0.2) | 45.6 (0.17) |
| Doctor Visit, % ## | Indicator of Hypertension knowledge and control | 1 (Yes), 0 (No) | 85.2 (1.0) | 85.5 (1.0) | 87.4 (0.8) | 86.1 (0.7) | 85.4 (0.9) | 72.6 (0.2) | 73.7 (0.2) | 74.7 (0.2) | 69.9 (0.2) | 70.5 (0.2) | 71.1 (0.16) |

Values in parentheses are standard errors, which account for survey design (calculated using R survey library).

* All estimates are age-standardized to the 2000 US population and incorporate the complex survey design for each survey, respectively.

** BRFSS estimates are presented for odd years between 1999 and 2009 when the “Hypertension Awareness” module was asked in all States for national comparability.

ƚ Measured hypertension unavailable in the BRFSS.

ǂ Medication information was not asked in any states in the BRFSS for this period.

# Smoking status determined by individual’s answer to the question “Have you smoked at least 100 cigarettes in your life?” which is asked in both surveys throughout the study period.
## This variable is calculated as a composite of other variables related to contact with a health professional in the past year for BRFSS years 2001-2004, since during these years this question was either not asked at all or only asked in optional modules in a subset of states.
