# Supplementary material for: Prevalence, Awareness, Treatment, and Control of Hypertension in United States Counties, 2001–2009
Source: PLoS One. 2013 Apr 5;8(4):e60308. doi: 10.1371/journal.pone.0060308 (PMC3618269; doi:10.1371/journal.pone.0060308)
Supplement: Table S4 — Coefficients for NHANES Predictive Models, Never Diagnosed Men and Women. (DOCX) [file pone.0060308.s010.docx]

Table S4: Coefficients for NHANES Predictive Models, Never Diagnosed Men and Women

|  | Men | | Women | |
| --- | --- | --- | --- | --- |
|  | Estimate | SE | Estimate | SE |
| Age | 0.05*** | 0.02 | 0.17*** | 0.03 |
| Age Squared | 0 | 0 | 0*** | 0 |
| Race |  |  |  |  |
| Non-Hispanic black | 0.77*** | 0.12 | 0.65*** | 0.15 |
| Hispanic | 0.01 | 0.12 | 0.1 | 0.14 |
| Other | 0.04 | 0.27 | 0.31 | 0.28 |
| BMI | 0.05*** | 0.01 | 0.02** | 0.01 |
| Health insurance | -0.27** | 0.13 | -0.28* | 0.15 |
| Education |  |  |  |  |
| Less than high school | 0.21* | 0.12 | -0.09 | 0.14 |
| More than high school | -0.16 | 0.12 | -0.28** | 0.13 |
| Doctor visit | -0.32*** | 0.11 | -0.8*** | 0.14 |
| Smoked 100 Cigarettes | -0.04 | 0.09 | -0.11 | 0.1 |
| Intercept | -6.06*** | 0.51 | -9.26*** | 0.79 |

***p-value < 0.01, **p-value<0.05, *p-value<0.1
