# Supplementary material for: Prevalence, Awareness, Treatment, and Control of Hypertension in United States Counties, 2001–2009
Source: PLoS One. 2013 Apr 5;8(4):e60308. doi: 10.1371/journal.pone.0060308 (PMC3618269; doi:10.1371/journal.pone.0060308)
Supplement: Table S5 — Coefficients for NHANES Predictive Models, Previously Diagnosed Men and Women. (DOCX) [file pone.0060308.s011.docx]

Table S5: Coefficients for NHANES Predictive Models, Previously Diagnosed Men and Women

|  | Men | | Women | |
| --- | --- | --- | --- | --- |
|  | Estimate | SE | Estimate | SE |
| Age | 0.09*** | 0.03 | 0.11*** | 0.03 |
| Age Squared | 0* | 0 | 0** | 0 |
| Race |  |  |  |  |
| Non-Hispanic black | 0.39*** | 0.12 | 0.33*** | 0.11 |
| Hispanic | 0.24* | 0.13 | -0.04 | 0.12 |
| Other | 0.32 | 0.33 | 0.02 | 0.26 |
| BMI | -0.01 | 0.01 | 0 | 0.01 |
| Health insurance | -0.55*** | 0.18 | -0.07 | 0.15 |
| Education |  |  |  |  |
| Less than high school | 0.03 | 0.13 | 0.2* | 0.11 |
| More than high school | -0.17 | 0.12 | -0.06 | 0.11 |
| Doctor visit | -0.08 | 0.25 | -0.38 | 0.26 |
| Smoked 100 Cigarettes | -0.15 | 0.1 | -0.12 | 0.09 |
| Medication use | -0.34* | 0.19 | 0.08 | 0.19 |
| Medication use*(2001-2002) | -0.32* | 0.17 | -0.14 | 0.15 |
| Medication use*(2003-2004) | -0.34* | 0.17 | -0.45*** | 0.15 |
| Medication use*(2004-2006) | -0.61*** | 0.17 | -0.77*** | 0.16 |
| Medication use*(2007-2008) | -0.58*** | 0.15 | -0.9*** | 0.14 |
| Intercept | -2.83*** | 0.95 | -4.27*** | 0.89 |

***p-value < 0.01, **p-value<0.05, *p-value<0.1
