# Supplementary material for: Prevalence, Awareness, Treatment, and Control of Hypertension in United States Counties, 2001–2009
Source: PLoS One. 2013 Apr 5;8(4):e60308. doi: 10.1371/journal.pone.0060308 (PMC3618269; doi:10.1371/journal.pone.0060308)
Supplement: Table S6 — Uncontrolled hypertension prevalence in NHANES and (imputed) BRFSS surveys by year and sex, age-standardized to 2000 US Population with 95% confidence intervals. (DOCX) [file pone.0060308.s012.docx]

Table S6: Uncontrolled hypertension prevalence in NHANES and (imputed) BRFSS surveys by year and sex, age-standardized to 2000 US Population with 95% confidence intervals.

|  | NHANES | | | | BRFSS | | | |
| --- | --- | --- | --- | --- | --- | --- | --- | --- |
|  | 2001-2002 | 2003-2004 | 2005-2006 | 2007-2008 | 2001 | 2003 | 2005 | 2007 |
| Men |  |  |  |  |  |  |  |  |
| Never Diagnosed | 0.10  0.09-0.12 | 0.08  0.06-0.10 | 0.10  0.08-0.13 | 0.11  0.09-0.12 | 0.11  0.09-0.12 | 0.11  0.10-0.12 | 0.11  0.10-0.13 | 0.12  0.11-0.13 |
| Previously Diagnosed | 0.22  0.18-0.26 | 0.23  0.19-0.27 | 0.24  0.20-0.28 | 0.21  0.18-0.25 | 0.29  0.24-0.34 | 0.26  0.23-0.30 | 0.23  0.21-0.25 | 0.22  0.20-0.25 |
| Women |  |  |  |  |  |  |  |  |
| Never Diagnosed | 0.17  0.13-0.20 | 0.13  0.10-0.15 | 0.11  0.09-0.14 | 0.11  0.09-0.13 | 0.15  0.13-0.16 | 0.14  0.13-0.16 | 0.15  0.13-0.16 | 0.14  0.13-0.16 |
| Previously Diagnosed | 0.32  0.28-0.36 | 0.32  0.29-0.35 | 0.26  0.22-0.29 | 0.22  0.20-0.25 | 0.36  0.33-0.40 | 0.32  0.28-0.36 | 0.28  0.25-0.32 | 0.26  0.24-0.29 |
