# Supplementary material for: Prevalence, Awareness, Treatment, and Control of Hypertension in United States Counties, 2001–2009
Source: PLoS One. 2013 Apr 5;8(4):e60308. doi: 10.1371/journal.pone.0060308 (PMC3618269; doi:10.1371/journal.pone.0060308)
Supplement: Appendix S1 — (DOCX) [file pone.0060308.s013.docx]

**Appendix S1**

**Small Area Model**

The model used for small area estimation is given by

$$\begin{matrix} Y_{ijt}\sim Bin(n_{ijt},p_{ijt}) \\ \log\left(\frac{p_{ijt}}{1-p_{ijt}}\right)=\alpha_{jt}+\beta_{i}+\gamma\cdot t \end{matrix}$$

Where

$Y_{ijt}$ is the number of individuals in Age-Race group *i*, county *j*, and year *t* with the outcome of interest,

$n_{ijt}$ is the number of individuals in Age-Race group *i*, county *j*, and year *t*,

$p_{ijt}$ is the prevalence of the outcome of interest in Age-Race group *i*, county *j*, and year *t*,

$\alpha_{jt}$ is the varying intercept for county *j* and year *t*,

$\beta_{i}$ is the fixed effect intercept shift for Age-Race group *i*,

$\gamma$ is the fixed effect slope for year *t*.

County-level variation is captured by the varying intercept, α_jt_, which is modeled as a function of county-level covariates and random effects. We considered four classes of model for the varying intercept, α_jt_, which we refer to as the Naïve, Geo-spatial, Covariate, and Full models. These are outlined below,

Naïve: random intercept for county.

$$\alpha_{jt}=\alpha_{0}+b_{0j}+b_{1j}t$$

Geo-Spatial: random intercept for county and geo-spatial covariate calculated as the neighborhood average of estimated random intercepts from Naïve model.

$$\alpha_{jt}=\alpha_{0}+b_{0j}+b_{1j}t+\delta\cdot\bar{b}_{j}^{post}$$

Covariate: county-level covariates with a random intercept for county.

$$\alpha_{jt}=\alpha_{0}+X_{ij}\theta+b_{0j}+b_{1j}t$$

Full: county-level covariates with random intercept for county and geo-spatial covariate calculated as the neighborhood average of estimated random intercepts from Covariate model.

$$\alpha_{jt}=\alpha_{0}+X_{ij}\theta+b_{0j}+b_{1j}t+\delta\cdot\bar{b}_{j}^{post}$$

Above, we have

$\alpha_{0}$is the fixed effect intercept,

$b_{0j}$is the random intercept for county *j*, with an assumed Gaussian distribution with mean zero, and variance $\sigma_{0}^{2}$,

$b_{1j}$is the random slope on time for county *j*, with an assumed Gaussian distribution with mean zero, and variance $\sigma_{1}^{2}$,

$\delta$ is the fixed effect of the neighborhood average of random intercepts from either the Naïve or Covariate models, $\bar{b}_{j}^{post}$,

$\theta$ is the vector of fixed effects for the county-level covariates, $X_{ij}$.

This taxonomy of models is designed to allow for the user to sequentially investigate the value added of including variables of different classes into the model.

**Model Selection and Validation**

Model selection and validation was performed by comparing county-level predictions to a pooled gold standard. The gold standard was defined as the age-standardized direct estimate of county-specific self-reported hypertension using all data pooled across the period 2001-2009. To construct a “validation set”, we identified counties with sample size of 900 or more observations over the periods 1997-2005 and 2001-2009. This resulted in 59 counties for men and 124 counties for women.

Counties in the validation set were randomly and repeatedly sampled down (10 times) to 10, 50, and 100 observations during the period 1997-2005. The small area models were then fit to the sampled down data from this period and age standardized predictions for 2005 were compared to the gold standard. We calculated the root mean squared error (RMSE) and concordance correlation (CORR) as a means of quantifying error and the final model was chosen on this basis. We compared the values of RMSE and CORR across the four outlined models above to determine a) which was the best performing model, and b) what the likely performance of the model was for sparsely populated counties.

The RMSE and CORR for the Naive, Geo-spatial, Covariate, and Full models are presented in Figure 1. Note that the results presented in the main article correspond to the Full model results in this figure. When looking across the four models in the small area model taxonomy, the Full model incorporating county-level covariates and geo-spatial information tended to show the most consistent performance. This is most notable in the extreme case where counties in the validation set were sampled down to only 10 observations each. In this case, we see a rather marked increase in the concordance correlation (CORR) between the gold standard and model predictions as we move from the Naïve to Full models. The CORR increases from 0.14 to 0.66 for men and from 0.33 to 0.84 for women. RMSE decreases from 0.038 to 0.022 for men and from 0.032 to 0.022 for women.

The difference between the Naïve, Geo-spatial, Covariate, and Full models tended to decrease as the sampling level increased from 10 to 100. This indicates that where data are prevalent, the model predictions are less sensitive to the model specification. However, where data are sparse, we anticipated the Full model to yield the most precise and least biased estimates.
